# A Subacute Presentation of Isolated Tuberculous Septic Hip Arthritis

**DOI:** 10.7759/cureus.43493

**Published:** 2023-08-14

**Authors:** Mustafa A. Al-Tikrity, Abdelaziz Mohamed, Ahmed K. A. Yasin, Nusiba H Elamin, Anas Mohamed

**Affiliations:** 1 Internal Medicine, Hamad Medical Corporation, Doha, QAT; 2 Internal Medicine, Hamad General Hospital, Doha, QAT

**Keywords:** anti-tb, tuberculosis (tb), hip joint, tuberculous septic arthritis, subacute

## Abstract

Tuberculous septic arthritis is a rare type of septic arthritis that is caused by *Mycobacterium tuberculosis*. However, it can lead to devastating complications if not diagnosed and treated correctly. We hereby report a 41-year-old female with no medical history who presented with a three-week history of right hip pain and inability to bear weight, found to have moderate to severe tenderness at the right anterior hip and gluteal area and limitation of joint movement. Magnetic resonance imaging (MRI) of the hip showed features of right hip septic arthritis with synovitis and anteromedial and posteromedial small collections. She was diagnosed with tuberculosis (TB) after joint fluid aspiration, and she was started on anti-TB treatment including isoniazid, rifampicin, ethambutol, and pyrazinamide directly after. Considering the case and the subacute presentation that can mimic bacterial septic arthritis, clinicians should always consider TB infection in their differential diagnosis upon assessing a suspected patient with septic arthritis even with a subacute presentation to achieve the correct diagnosis and start appropriate treatment to avoid its harmful complications.

## Introduction

Tuberculosis (TB) is a bacterial infection that is usually caused by *Mycobacterium tuberculosis*. However, a small number of cases are caused by other mycobacterial species. According to the World Health Organization, TB killed 1.6 million people in 2021. It was the 13th leading cause of death worldwide and the second leading infectious killer after COVID-19 [1]. Although TB most usually impacts the lungs, it can affect various other organs, otherwise known as extrapulmonary TB.

Tuberculosis can infect the joints, causing tuberculous septic arthritis. Of extrapulmonary TB cases that affect the musculoskeletal system, 15%-20% include the hip [2]. TB infection of the musculoskeletal system occurs from the reactivation of the mycobacterium that reaches the joint or the bone through the bloodstream that can enter the joint space initiating the inflammatory process [3]. This disease has a wide range of presentations that include asymptomatic patients with a normal radiographic examination to severe cases of joint pain along with joint destruction, osteomyelitis, or abscess formation. Most symptomatic cases of TB hip arthritis are presented with pain and joint movement restriction of the hip. Therefore, the diagnosis can be challenging because it is often mistaken for other diseases with similar symptoms [4]. It usually presents a high risk of deterioration and destruction of the joints involved. Therefore, the delay in the diagnosis might lead to poor outcomes for the joint. Clinicians assessing patients presenting with joint pain, swelling, joint movement restriction, and/or inability to bear weight might have difficulty in early diagnosis of this disease due to its similarity with other common cases of septic arthritis.

The treatment regimens for TB arthritis includes the four anti-TB medications isoniazid, rifampicin, pyrazinamide, and ethambutol, which are typically prescribed with further follow-up of sensitivity results to determine drug resistance result. In some cases, surgical interventions may be needed to treat joint destruction and relieve patient symptoms [5]. The prognosis of the disease depends on its stage and progression at presentation along with proper diagnosis and early management [4]. We hereby report a case of an acute presentation of right tuberculous hip monoarthritis that presented with a three-week history of right hip pain and inability to bear weight, diagnosed with TB after joint fluid aspiration and started on anti-TB treatment directly after.

## Case presentation

A 41-year-old female with no medical history of diseases presented to the emergency department (ED) with three weeks of right hip pain, 6/10 on the visual analog scale, radiating to the thigh and knee. She also reported an inability to bear weight for five days.

Upon further history taking, she denied any history of fevers, cough, weight loss, trauma, fall, travel, or recent other infection(s). The patient reported having lower back pain three months ago that resolved on its own after two days. She has no history of other joint involvement, viral infections, UTI, or diarrhea. The patient did not have any significant medical, surgical, or family history of diseases.

During the assessment in the emergency department, she was uncomfortable due to the pain. However, she was hemodynamically stable. Her blood pressure was 138/84 mmHg, pulse rate 88 bpm, respiratory rate 16 breaths/minute, and temperature 36.9°C. Upon local examination, there was no obvious deformity, and she had moderate to severe tenderness at the right anterior hip and gluteal area that limited the range of motion in all directions. There were tender multiple inguinal lymph nodes. There was mild to moderate tenderness on the lower spine and right lumbar areas. Other general and systematic examinations were unremarkable, including the chest.

The patient underwent the needed investigations, including blood and radiological tests. The findings were as follows: white blood cell (WBC), 4.6 × 10^9^/L; hemoglobin (Hb), 9.8 q/dL; platelets, 400; C-reactive protein (CRP), 29 mg/dL; procalcitonin, 0.03 ng/mL; Na, 137 mmol/L; and K, 2.9 mmol/L.

**Figure 1:**
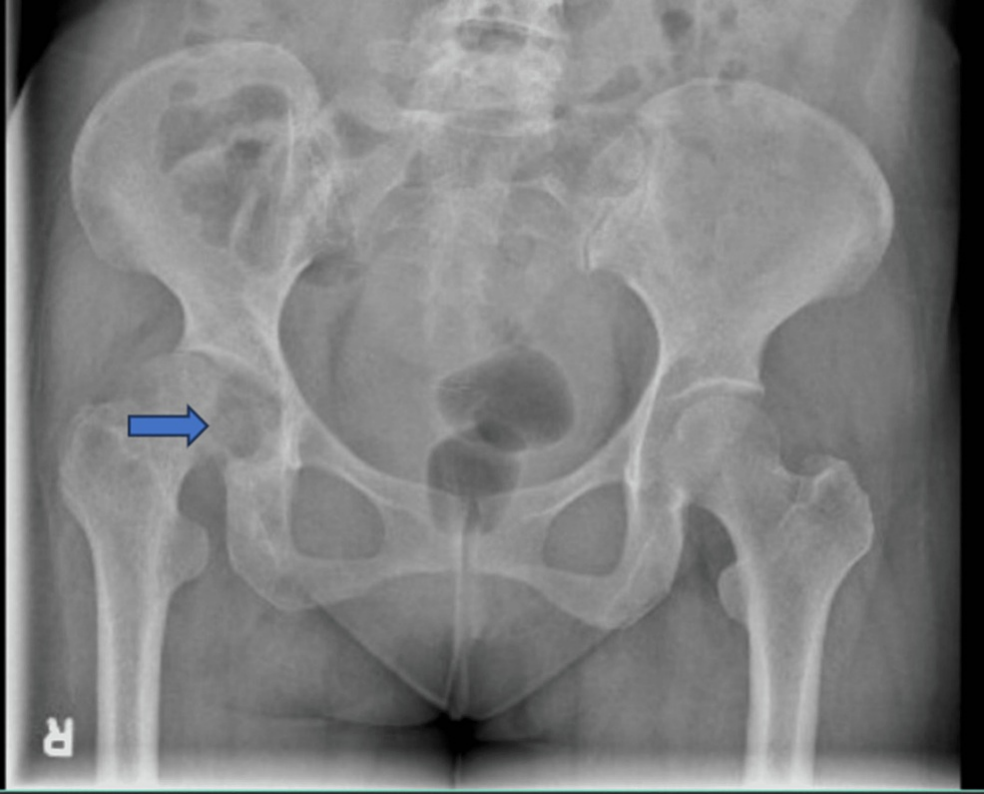
X-ray (anteroposterior view) of the pelvis with both hip joints showing lytic lesion in the right acetabulum (blue arrow).

**Figure 2:**
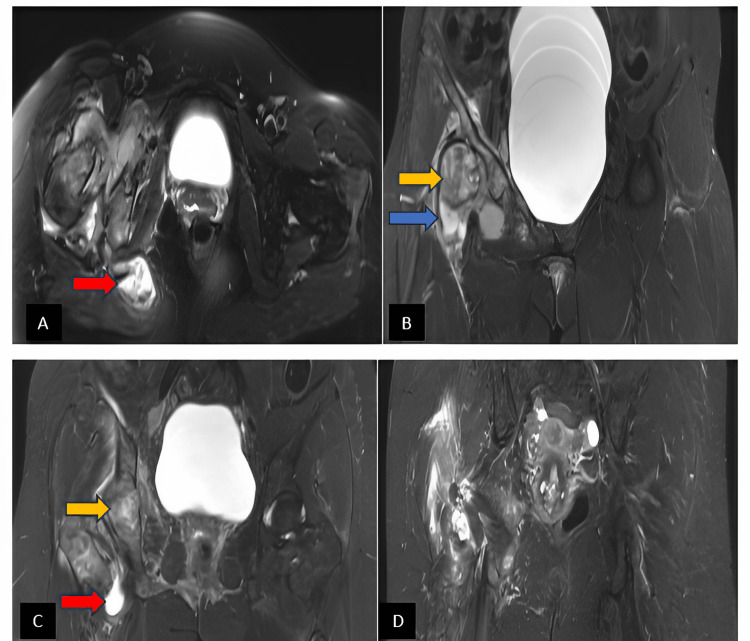
MRI of the right hip with contrast: axial view in T2WI (A), coronal view in T2WI (B), T2WI with contrast (C), and T2W1 of the right hip (D). There is effusion with synovial thickening of the right hip showing enhancement after contrast (blue arrow) and diffuse bone marrow edema involving the right acetabulum (yellow arrow). A large focus anterior to the right gluteus maximus muscle medially measuring 3.5 × 2.7 cm shows increased signal intensity on T2 with another focus adjacent to the right acetabular labrum measuring 2.8 × 1.7 cm slightly hyperintense on T2, representing collections (red arrow). MRI: magnetic resonance imaging, T2WI: T2-weighted image

X-ray of the right hip showed a lytic lesion in the acetabulum (Figure 1). Accordingly, magnetic resonance imaging (MRI) was performed. It showed a large effusion with synovial thickening of the right hip showing enhancement representing active synovitis with inflammatory effusion and diffuse bone marrow edema involving the right acetabulum, femoral head, and proximal femur with early avascular necrosis on the medial surface of the hip joint. Also, there was a large focus anterior to the right gluteus maximus muscle medially measuring 4.5 × 2.7 cm with another focus adjacent to the right acetabular labrum measuring 2.8 × 1.7 cm isointense on T1 but slightly hyperintense on T2, likely representing collections (Figure 2).

The patient was admitted to the hospital, and computed tomography (CT)-guided aspiration of the right hip/buttock collection was performed. During the procedure, 7 mL of dark brownish fluid was aspirated and sent to the laboratory for microbiological investigation. At the same period, the patient was treated conservatively with analgesia and ceftriaxone IV 2 g/day. The aspiration results showed the presence of acid-fast bacilli (AFB) on the smear (3 AFB/100 fields) and tuberculosis (TB) DNA by polymerase chain reaction (PCR). The infectious disease team was involved and requested a chest X-ray and sputum AFB for her. The chest X-ray showed no abnormalities, and the two sputum smears for acid-fast bacilli were negative.

The patient was started on anti-TB medication including isoniazid, rifampicin, pyrazinamide, and ethambutol with a plan to treat for 9-12 months guided by the response and the culture's susceptibility. Cultural susceptibility showed no resistance. The orthopedic team reviewed the patient's case and agreed to a conservative approach, as she planned to travel abroad for further treatment, with gradual weight-bearing and follow-up. After three days, the patient was discharged from the hospital. The patient's discharge assessment showed that she was able to ambulate independently with a mild limp and continue her care with a physician and orthopedic surgeon abroad.

## Discussion

Tuberculous arthritis can infect patients even without a history of TB infection, immunocompromised status, or contact with patients who have or had TB. Sometimes, those patients might get the infection during their exposure to undiagnosed cases of TB.

The patient in this case report presented with isolated right hip TB arthritis without pulmonary or other common extrapulmonary involvement such as the spine. Physicians should always consider TB as one of the major causes of primary septic arthritis in their differential diagnosis when assessing those cases especially when the patient is present with slow-progressing symptoms or has recurrent ED visits.

The usual presentation of TB arthritis includes minimal and slow symptoms that build up over months that delay its diagnosis. Symptoms of fever, night sweats, weight loss, or others are usually absent or mild if present. Local joint symptoms appear as pain mainly affecting ambulation, and it might be accompanied by stiffness and deterioration in joint function over months [4]. It has been reported that the mean delay until the diagnosis of tuberculous arthritis may be as long as two months [6]. Our patient was presented with three weeks of right hip pain and inability to bear weight, which is not a usual presentation to have unless superadded bacterial infection occurs.

Reviewing the literature reveals a few reported cases of TB arthritis of the hip joints, and all are of monoarthritis type. A case report from Saudi Arabia described a 15-year-old female patient who presented with chronic hip pain over months that led to an inability to bear weight, and the patient was later found to have TB arthritis [7]. Another reported case is of an 81-year-old female diagnosed with septic arthritis superinfected with methicillin-resistant *Staphylococcus aureus* (MRSA) and *Mycobacterium tuberculosis* simultaneously, which was treated with antibiotics and surgical drainage [8].

Diagnosing TB arthritis should be made using clinical assessment, radiological changes, culture of the aspirated fluid, and/or histopathologic assessment of biopsy specimens. X-ray as an initial radiographic modality has a very low yield with many cases appearing with unremarkable bone structure, and most changes will appear in advanced and late disease presentation affecting the bone. One of the reported classic radiographic presentations on X-ray in advanced cases includes the Phemister triad: periarticular osteoporosis, osseous erosion on peripheral distribution, and gradual narrowing of the joint space [9].

Magnetic resonance imaging (MRI) of the hip joint provides very useful information in the diagnosis and differentiation between tuberculous arthritis and pyogenic arthritis. Hong et al. mentioned that findings of bone erosion are more in TB arthritis; on the other hand, subchondral marrow signal intensity abnormality was more in patients with pyogenic arthritis [10]. MRI findings of synovitis, effusion, central and peripheral erosions, active and chronic pannus, abscess, bone chips, and hypointense synovium suggest and favor tuberculous cause [11].

Early treatment should be initiated as soon as the diagnosis of TB septic arthritis is confirmed. Using standard chemotherapy with four anti-TB medications is accepted in all stages or presentations of the disease [12]. The duration of the anti-TB therapy ranges from six to nine months and can be extended to 12 months or even longer in patients with extensive or advanced disease [12]. The infectious disease team of our patient has decided to go with a 9- to 12-month treatment regimen and to follow the response and the culture's susceptibility.

Skeletal traction has been also recommended to most patients, works to relieve muscle spasms, maintains the joint space area, and corrects deformity and subluxation [5]. In the early stage of the disease, if the patient did not improve on the conservative treatment, then a surgical approach with either joint debridement and synovectomy with follow-up of the response or total hip arthroplasty may be performed. For severe TB arthritis, surgery may be necessary to remove damaged tissue from the hip joint [5]. The joint will then be immobilized in a spica cast for 6-8 weeks for better healing. Rehabilitation will follow to help the patient regain range of motion and strength. If the functional results are still not acceptable after adequate treatment, the patient may be a candidate for other surgical options, such as resection arthroplasty or total hip arthroplasty (THA), depending on the extent of damage to the joint [5].

## Conclusions

Isolated tuberculous hip arthritis is one of the uncommon presentations of extrapulmonary TB that can develop without prior TB history or immunocompromised status. Although the typical symptoms of tuberculous arthritis, which progress slowly over months, may not be present in all cases, a subacute presentation should still raise suspicion for tuberculosis in the differential diagnosis. Magnetic resonance imaging (MRI) is a useful tool for diagnosing and differentiating between tuberculous arthritis and pyogenic arthritis. Prompt treatment with antituberculosis medications is essential after diagnosis. Skeletal traction can be used to help reduce pain and inflammation, with a surgical approach with either joint debridement and synovectomy or total hip arthroplasty needed in most of the presentations.
